# Effects of a web-based education for community mental health case managers on physical healthcare for clients with severe mental illness

**DOI:** 10.3934/publichealth.2023045

**Published:** 2023-08-04

**Authors:** Jungsu Lee, Yun-Jung Choi

**Affiliations:** 1 Seoul Psychiatric Emergency Response Center, Seoul, South Korea; 2 Chung-Ang University, Red Cross College of Nursing, Seoul, South Korea

**Keywords:** case management, community mental health, nursing education, physical health care, web-based learning

## Abstract

This study aimed to develop and verify the effects of a web-based physical healthcare education program for community mental health case managers during the time of COVID-19. Six modules of mental health case management physical health education program were developed and provided using the EdWith education platform, which enables real-time streaming, lecture participant management and whether participants have watched the video and watch time. A total of 51 community mental health case managers participated in the study. Collected data were analyzed using SPSS 26.0 software. Participants of the physical healthcare education program testified increased performance in, as well as enhanced attitudes toward physical healthcare. Their confidence in physical healthcare increased significantly from that of the individuals in the control group. The web-based educational program for mental health case managers in physical healthcare may be beneficial to improving the physical health of clients with chronic mental illness.

## 1. Introduction

People who experience severe mental illness have a shorter life expectancy than the general population [1]–[3]. Death due to suicide accounts for a high proportion of the short life expectancy of people with mental illness, but physical diseases such as cardiovascular disease, cancer and other chronic diseases are also cited as causes [4]–[5]. Sixty-five percent of patients with schizophrenia have one or more chronic physical diseases [6]. In particular, those with mental illness have a 10%–24% higher risk of cardiovascular disease than the general population, and metabolic syndrome occurs two to three times more [6],[7]. This is related to overweight, dyslipidemia and smoking habits, which are common in mentally ill patients [7]. Although the risk of physical health is high, mentally ill people are experiencing difficulties in using medical services such as medical examinations and medical treatment related to physical health [8].

In developed countries, mental health case managers regularly evaluate physical health, and in connection with hospitals, disease prevention and treatment are provided through health checkup services [9]. In this process, the ability of the mental health case manager to assess the physical health of the mentally ill at an early stage and link it to a treatment institution is important [10]. Nevertheless, the problem-solving ability of community mental health case managers for physical health management is low [11]. Mental health case managers perceive that their physical health management competency is lower than the required level, which is indicated by a high education requirement for physical health management [12].

However, there is a lack of physical health education for mentally ill patients targeting mental health case managers [13]. Although the importance of physical health management for mentally ill people is increasing, it is necessary to develop and provide education for mental health case managers because there is insufficient education or support for physical health management methods. Therefore, this study was conducted with the purpose of confirming the effectiveness of developing a web-based physical health management education program for patients with chronic mental illness for mental health case managers.

## 2. Materials and Methods

### 2.1. Study design

The study applied a non-equivalent control group pre and posttest design to examine the effects of a web-based physical health care education program for mental health case managers.

### 2.2. Study Participants

The subjects of this study were mental health case managers working in community mental health organizations. In Korea, community mental health case managers are affiliated with mental health welfare centers established by each administrative district to provide community mental health case management. Study participants were recruited from a social network of community mental health case managers who understood the purpose and contents of this study and voluntarily participated in the web-based physical health management education program for chronic mental illness patients. The number of study subjects was calculated using G* Power 3.1 with a significance probability of 0.05, an effect size of 0.80 and a power of 0.80 as a minimum of 52. The study participants were recruited using the case manager online community bulletin board and community mental health centers. A total of 60 participants were finally selected in consideration of dropouts and insufficient questionnaires.

### 2.3. Study Procedure

The educational program of this study was developed based on the ADDIE model, an acronym for the five stages of a development process: Analysis, Design, Development, Implementation and Evaluation (Table 1) [14].

**Table 1.** Development processes of the Web-based Education Program using the ADDIE Model.

**Step**	**Development Processes of the Web-based Education Program**
**Analysis**	Literature review on physical healthcare for clients with severe mental illness. Learner needs analysis by individual interviews with community mental health case managers.
**Design**	Set learning goals of the web-based education program. Organize educational content on physical healthcare for clients with severe mental illness. Learning method design for community mental health case managers. Plan for measuring effectiveness of the web-based education program.
**Development**	Production of the educational materials of the web-based education program. Verification of the educational contents on physical healthcare for clients with severe mental illness. Video production for online transmission for community mental health case managers.
**Implementation**	Recruit learners and provide orientation of the web-based education program. Providing an online streaming education environment using the EdWith platform.
**Evaluation**	Performance in physical health care. Attitudes toward physical health care. Confidence in physical health care.

In the analysis stage, learner interviews and literature review were conducted to investigate learner characteristics, learner jobs and tasks, learner needs and educational environment. As a result, the education contents were required to physical health care management competence including knowledge about cardiovascular disease, endocrine disease and metabolic syndrome. The level of education was needed at a level that can be easily understood by non-medical professionals such as social workers and clinical psychologists. As for the education method, it was analyzed that web-based online education with free time and space was appropriate considering the COVID-19 situation.

Based on this analysis, a mental health case management physical health education program consisting of a total of six modules was developed. It takes fifty minutes for the case managers to complete the web-based program. The final education program was completed after receiving content validity evaluation and expert opinions from a total of 5 experienced mental health case managers (Table 2).

**Figure 1.**
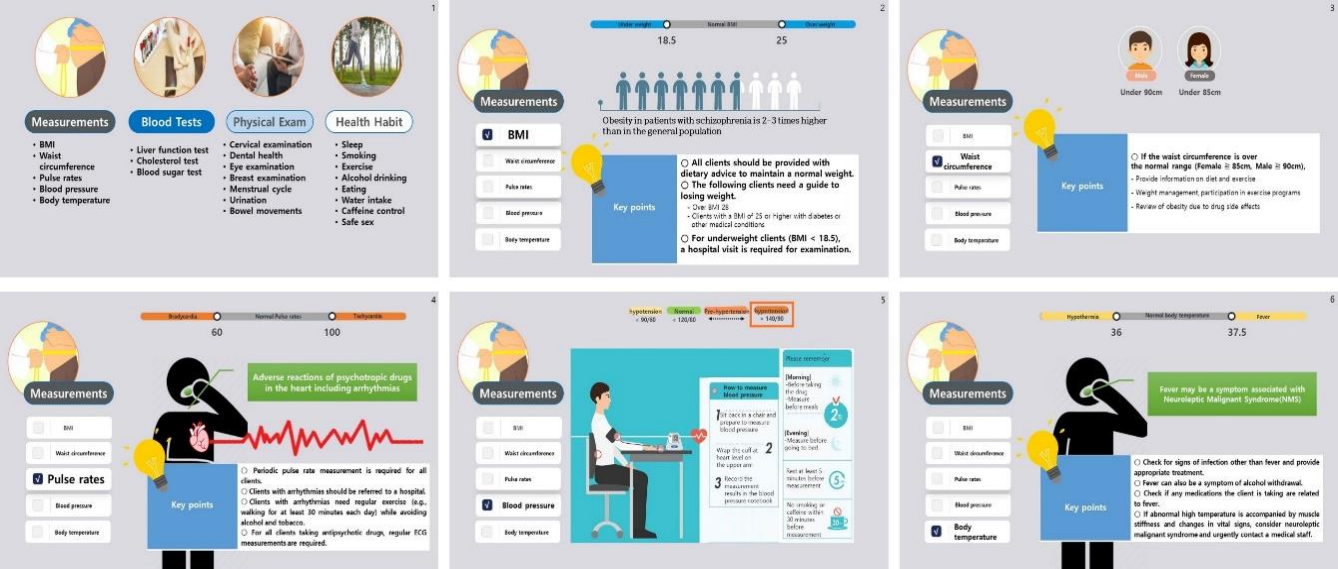
Web-based physical healthcare education program.

**Figure 2.**
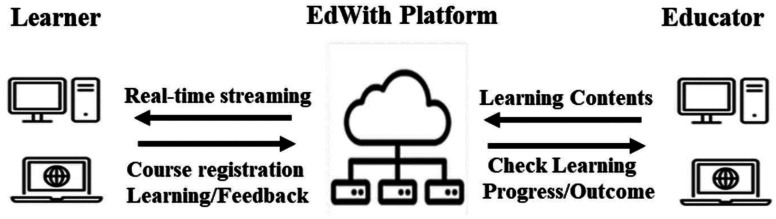
System interface using the EdWith platform.

Figure 1 provides examples of the education contents. The completed educational program was provided using the EdWith education platform, which enables real-time streaming. The educator can use this platform to a create learning course and invite learners or accept requests from learners to participate in the lecture. This platform provides the educator with the function to check the learner's learning progress and learning time so that the educator can check the learning status of the learners. (Figure 2).

**Table 2.** Web-based physical healthcare education program for mental health case managers.

**Summary of the Learning Contents**		**Time**
**Session 1.** Necessity of physical healthcare for clients with chronic mental illnesses.		15 min
**Module 1.** General physical health conditions of clients with chronic mental illnesses	**Learning Goal:** Understand the physical health care needs of clients with chronic mental illness. **Learning Overview:** Session 1 consists of two module video lectures to introduce the physical health status of clients with chronic mental illness and to recognize their needs for physical health management. Each lecture provides details about the physical health vulnerabilities and care needs of people with chronic mental illness.	
**Module 2.** Physical health care for clients with chronic mental illnesses: (1) Physical activity, (2) Diet, (3) Alcohol drinking, (4) Smoking, (5) Sex life, (6) Health beliefs		
**Session 2.** Physical healthcare checklist for clients with chronic mental illnesses		35 min
**Module 3.** Measurements: (1) BMI, (2) Waist circumference, (3) Pulse rates, (4) Blood pressure, (5) Body temperature	**Learning Goal:** Utilize the physical health care checklist in mental health case management based on an actual understanding of it. **Learning Overview:** Session 2 consists of four module video lectures for utilizing the physical health management checklist for clients with chronic mental illness. In each module, education on the concept and assessment of each checklist item, interpretation of evaluation and follow-up care was provided. Mental health case managers can utilize the physical health management checklist learned in this lecture to evaluate the overall physical health status of patients with chronic mental illness and provide necessary help appropriately.	
**Module 4.** Blood tests: (6) Liver function, (7) Cholesterol, (8) Glucose		
**Module 5.** Physical examination: (9) Cervical examination, (10) Dental health, (11) Eye examination, (12) Breast examination, (13) Menstrual cycle, (14) Urination, (15) Bowel movements		
**Module 6.** Health habits: (16) Sleep, (17) Smoking, (18) Exercise, (19) Alcohol drinking, (20) Eating habit, (21) Meal Prep and Cooking, (22) Water intake, (23) Caffeine control, (24) Safe sex		

**Total**		**50 min**

### 2.4. Study Instruments

#### 2.4.1. Performance in physical health care

To measure the level of Performance in physical health care of participants in this study, “Current practice in physical health care questionnaire” was used. This tool was developed to evaluate the physical health care activities of case managers [15]. In this study, a modified Korean language tool was used for the study of measuring the physical health nursing performance of Korean clinical nurses. This tool consists of a total of 13 questions and a 5-point Likert scale, where a higher score means a higher level of physical health care activity. Examples of items in this instrument include “I can measure the waist circumference of the client” and “I can measure the blood pressure of the client”. The reliability of the tool in this study was Cronbach's α = 0.90, indicating the same level of reliability as the previous study conducted on Korean clinical nurses [16],[17].

#### 2.4.2. Attitudes toward physical health care

The participants' levels of attitudes toward physical health care in this study were measured by “Attitudes toward physical health care” among the subscales of “Physical Health Attitude Scale for mental health nurses (PHASe)” [15]. In this study, the tool translated into Korean and modified a 5-point Likert scale consisting of 9 items to evaluate the attitudes toward physical health care level of community mental health professionals [17]. Examples of items measured by this instrument are “It is the mental health case manager's role to help clients manage their weight” and “It is the mental health case manager's role to educate clients to have regular eye exams.” In previous studies, the reliability of the tool was found to be Cronbach's α = 0.78 to 0.86 [16],[17], and the reliability in this study was found to be Cronbach's α = 0.87.

#### 2.4.3. Confidence in physical health care

The level of confidence in the physical health management of participants in this study was measured by the subscale “Confidence in providing physical health care” among “Physical Health Attitude Scale for mental health Nurses (PHASe)” [15]. In this study, a 5-point Likert scale consisting of 6 items was used to evaluate the confidence in the level of physical health care of community mental health professionals by modifying the tools translated into Korean [17]. Examples of items in this instrument include “I can assess the signs and symptoms of high blood sugar” and “I can perform BLS (basic life support) when a client has a heart attack.” The reliability of the tool was Cronbach's α = 0.74 in a previous study [17], and Cronbach's α = 0.86 in this study.

### 2.5. Data collection

The study participants were randomly assigned to the experimental group or control group and accessed the online URL sent via e-mail to perform pre-test from May 16 to 19, 2021. The post-test was conducted from May 28 to 31, 2021 after the end of the web-based training. In this study, data from a total of 52 participants were used for analysis among 60 pre-test respondents, excluding 3 people who did not complete training in the experimental group, 1 who did not submit a post-test in the experimental group and 4 who did not submit a post-test in the control group.

### 2.6. Data analysis

Collected data were analyzed using SPSS 26.0 program. The general characteristics of the subjects were analyzed as frequency, percentage, mean and standard deviation. The homogeneity test between the experimental group and the control group was analyzed by t-test, x² test and Fisher's exact test. Differences before and after the training program between the experimental group and the control group were analyzed using paired t-test and one-way ANOVA.

### 2.7. Ethical considerations

This study received approval from the Institutional Review Board of Chung-Ang University in Seoul, Korea. Considering that it is a non-face-to-face web-based education, explanations about the purpose of the study, confidentiality of data and disposal of data were provided to study participants via e-mail. After explaining that the study could be stopped at any time during the study and that there would be no disadvantages due to this, the written informed consent was obtained voluntarily to participate in the study. It was explained that the collected research data are not used for any purpose other than research, the data are stored separately in a secure place through an encryption process, and are discarded after 3 years of data storage. A small gift was given to the study participants.

## 3. Results

The gender of the participants in this study was 6 males (11.5%) and 45 females (88.5%). The professional qualifications of the participants consisted of 38 social workers (73.1%), 12 nurses (23.1%) and 2 clinical psychologists (3.8%). As for professional experience, 5–10 years were the most at 28.8%, 25% less than 3 years and 13.5% more than 15 years. There was no statistically significant difference between the experimental group and the control group as a result of the homogeneity test according to general characteristics, so it was confirmed that the two groups were homogeneous (Table 3).

**Table 3.** Homogeneity test of general characteristics between the two groups (*N* = 52).

Variables	Categories	Exp. (n = 26)	Cont. (n = 26)	*χ²* or *t*	*p*
		N (%)	N (%)		
Gender	Male	3 (11.54)	3 (11.54)	0.00	1.000
	Female	23 (88.46)	23 (88.46)		
Age	20–39	21 (80.77)	20 (76.92)	0.12	1.000
	40–60	5 (19.23)	6 (23.08)		
Education	Associate	0 (0)	1 (3.85)	1.60	0.450
	Bachelor	15 (57.69)	17 (65.38)		
	Master	11 (42.31)	8 (30.77)		
Professional qualifications	Social worker	19 (73.08)	19 (73.08)	2.33	0.311
	Nurse	5 (19.23)	7 (26.92)		
	Clinical psychologist	2 (7.69)	0 (0)		
Mental health professional	Yes	24 (92.31)	25 (96.15)	0.35	1.000
	No	2 (7.69)	1 (3.85)		
Workplace	MMHWC	11 (42.31)	10 (38.46)	5.44	0.245
	BMHWC	10 (38.46)	13 (50)		
	Social rehabilitation center	0 (0)	2 (7.69)		
	Alcohol rehabilitation center	3 (11.54)	1 (3.85)		
	Other community mental health service center	2 (7.69)	0 (0)		
Career (years of work experience)	<3	7 (26.92)	6 (23.08)	0.93	0.920
	≥3, <5	5 (19.23)	4 (15.38)		
	≥5, <10	6 (23.08)	9 (34.62)		
	≥10, <15	4 (15.38)	4 (15.38)		
	≥15	4 (15.38)	3 (11.54)		

Note: MMHWC = Metropolitan mental health welfare center; BMHWC = Basic mental health welfare center.

After the training program in the experimental group, the performance in physical health care score increased by 0.58 points compared to the pre-test, and there was a statistically significant difference (*t* = -3.767, *p* < 0.001), whereas the pre and post test scores of the control group didn't show no statistically significant difference. There was no statistically significant difference (*t* = -0.897, *p* = 0.378), and there was a statistically significant difference between the performance in physical health care scores before and after training of the experimental group and the control group (*F* = 7.115, *p* < 0.001).

The attitudes to physical health care score in the experimental group after the training program increased by 0.51 points compared to the score before the training program, and there was a statistically significant difference (*t* = -3.842, *p* < 0.001), while there was no significant difference between the pre and post scores of the control group (*t* = 1.703, *p* = 0.101). There was a statistically significant difference between the attitudes to physical health care scores before and after training of the experimental group and the control group (*F* = 5.776, *p* < 0.005).

After the training program in the experimental group, the confidence in physical health care score increased by 0.71 points compared to the score before the training program, and there was a statistically significant difference (*t* = -5.618, *p* < 0.001). However, the pre and post test scores of the control group didn't show no statistically significant difference (*t* = -0.891, *p* = 0.381). There was a statistically significant difference between the confidence in physical health scores before and after education of the experimental group and the control group (*F* = 5.479, *p* < 0.05) (Table 4).

**Table 4.** Comparison of dependent variables between the two groups after treatment (*N* = 52).

Variables	Group	Pre-test	Posttest	Paired *t*-test		*ANOVA*	
		*M ± SD*	*M ± SD*	*t*	*p*	*F*	*p*
Performance in physical healthcare	Experimental group (n = 26)	2.83 ± 0.48	3.41 ± 0.71	-3.77	0.001***	7.12	0.001***
	Control group (n = 26)	2.81 ± 0.68	2.86 ± 0.64	-0.90	0.378		
Attitudes toward physical healthcare	Experimental group (n = 26)	3.24 ± 0.48	3.75 ± 0.70	-3.84	0.001***	5.78	0.005**
	Control group (n = 26)	3.31 ± 0.71	3.20 ± 0.74	1.70	0.101		
Confidence in physical healthcare	Experimental group (n = 26)	3.12 ± 0.78	3.83 ± 0.84	-5.62	<.001***	5.48	0.006**
	Control group (n = 26)	3.01 ± 1.00	3.09 ± 1.08	-0.89	0.381		

Note: *p < 0.05, **p < 0.01, ***p < 0.001.

## 4. Discussion

This study was conducted to confirm the effectiveness of developing a web-based physical health management education program for chronic mental illness patients for mental health case managers.

In this study, there was an improvement in the case managers' perception of their ability to manage and address physical health conditions. This result is consistent with previous studies in which there was an improvement in meaningful knowledge before and after provision of physical health management education for mental health nurses and an improvement in the performance intention to be applied [10]. Therefore, it would be possible to increase the physical health management performance of mental health case managers through the provision of the educational program developed in this study, which will help the chronic mental illness patients in the community to use physical health services.

Mental health case managers' attitudes toward physical health care affects the actual performance of physical health care by making them recognize that physical health care is a task to be performed in providing case management [17]. In the same vein, in this study, mental health case managers significantly changed their physical health management attitudes before and after participating in the web-based education program. This means that the checklist items contributed to the recognition of mental health case managers as contents that should be checked for physical health management of mentally ill patients [18].

In general, it is known that patients with schizophrenia and bipolar disorder have less participation in physical activity [19]. In this study, the mental health case managers' perceptions of physical health care related to exercise also showed a low score. This recognition is thought to be an obstacle for the mental health case manager to manage the case for exercise [10]. In future education, it is necessary to emphasize the importance of exercise in physical health care and supplement the educational contents to recognize the importance of exercise for mentally ill people.

This study is important in that the physical health checklist used in this study consists of physical measurements, vital signs measurement, blood tests, physical examination and lifestyle, so it is a useful and convenient instrument that can be directly applied in the actual community mental health management field. Based on this, it provides guidelines for practitioners to check the physical health of those mentally ill by providing community mental health case managers with the items to check for mentally ill people, the normal range of physical health values and precautions for physical health management. In addition, it makes it possible to check the overall health without omission [18]. Therefore, by distributing the checklist developed in this study to mental health case management practitioners, it is expected that it will be helpful in promoting the physical health of chronic mental illness patients.

In this study, it was confirmed that the web-based education program was effective as the level of physical health care performance, attitude and confidence increased significantly. Web-based education can be taught without restrictions on time and place, can be used continuously as a training material in practice and has the advantage of contributing to standardization by providing the same educational content [20]. The educational program provided in this study will also be able to contribute to the standardization of physical health management by providing mental health case managers with the same physical health management checklist. In addition, web-based education allows learning without time and space constraints, and is useful as a method that has educational effects while allowing trainees to participate in the progress and content of their own learning [21]. Therefore, it is expected that the web-based physical health management education program developed in this study will be used by mental health case managers to contribute to the physical wellness of the community.

Limitations of this study include that this study was conducted in a short period of time targeting a relatively small group of participants, and case managers were not actually followed to see if there was a change in behavior, nor were service users evaluated to understand the impact. Those limitations require careful application of the results directly. It is recommended that long-term repeated studies with a larger and more diverse population be conducted in the future. In addition, knowledge assessment after learning was not provided in this study. It is recommended to include a knowledge assessment at the end of each module in further research. Lastly, this study did not confirm the effect of learning retention through follow-up evaluation. In future studies, it is suggested that a follow-up assessment after 3 months be conducted to confirm the learning retention after the web-based education.

## 5. Conclusions

This study is meaningful in that it developed a web-based education program for mental health case managers for physical health care of clients with chronic mental illness and verified its effectiveness. The educational program developed in this study provides physical health management tools in a checklist method and deals with methods for checking physical health statuses that can be widely learned and used. Although the effectiveness of the program was subjective as measured over a short period of time, this is thought to increase the level of physical health care performance of mental health case managers for chronic mental illness patients in the community because the educational contents can be directly applied to practice. This increase in educational opportunities is expected to enable mental health case managers to perform physical health care for chronic mental illness patients with a more active attitude and confidence.

## Use of AI tools declaration

The authors declare they have not used Artificial Intelligence (AI) tools in the creation of this article.
